# The Sexual and Reproductive Health of Adolescents with Cerebral Palsy in Rural Bangladesh: A Qualitative Analysis

**DOI:** 10.1007/s10508-023-02535-4

**Published:** 2023-01-24

**Authors:** Rosalie Power, Eamin Heanoy, Manik Chandra Das, Tasneem Karim, Mohammad Muhit, Nadia Badawi, Gulam Khandaker

**Affiliations:** 1 Translational Health Research Institute, Western Sydney University, Locked Bag 1797, Penrith South, NSW 2751 Australia; 2 Discipline of Child and Adolescent Health, Sydney Medical School, University of Sydney, Sydney, NSW Australia; 3 Asian Institute of Disability and Development, University of South Asia, Dhaka, Bangladesh; 4 CSF Global, Dhaka, Bangladesh; 5 Cerebral Palsy Alliance Research Institute, University of Sydney, Sydney, NSW Australia; 6 Central Queensland Public Health Unit, Central Queensland Hospital and Health Service, Rockhampton, QLD Australia; 7 School of Public Health, The University of Queensland, Brisbane, QLD Australia

**Keywords:** Cerebral palsy, Adolescent, Bangladesh, Sexual and reproductive health, Global south

## Abstract

Adolescents with disability in the Global South have unique sexual and reproductive health (SHR) experiences and needs; however, they are rarely included in SRH discourse. This qualitative study, conducted in rural Bangladesh, used semi-structured interviews to understand how adolescents with cerebral palsy (CP) experience their SRH. Participants were recruited from the Bangladesh Cerebral Palsy Register and included 24 adolescents with CP (*n* = 12 female; *n* = 12 male) and 76 parents (*n* = 56 mothers, *n* = 17 fathers, *n* = 3 other relatives). Data were analyzed using reflexive thematic analysis. Findings highlighted heterogeneity among adolescents with CP including differences for adolescent men versus women. For some adolescent men with CP, sexual maturity was viewed as bringing new opportunities, whereas for other men, adolescence affirmed exclusions and some transgressed sociocultural norms as they struggled to navigate their pubescent body alongside new privacy requirements. For adolescent women with CP, sexual maturity was associated with new domestic responsibilities, silence and secrecy regarding menstruation, and increased vulnerability to sexual violence and abuse. Adolescent men and women with CP spoke about marriage as something “everybody wants,” however, was deemed “impossible” for those with more impairment-related support needs. Both adolescent men and women with CP lacked access to SRH information and support. Mothers positioned providing care to their adolescent child with CP after puberty as “shameful.” Our findings suggest that disability, health, and education services in rural Bangladesh need to adopt a life-course approach that incorporates the SRH of adolescents with CP. We recommend the provision of SRH education that addresses the physical, cognitive, and social needs of adolescents with CP.

## Introduction

Adolescent sexual and reproductive health (SRH) was announced as having “arrived” in 1994 at the International Conference on Population Development (United Nations Population Fund, 1994). Considered pertinent to the everyday wellbeing of adolescents, the agenda was to embed SRH rights at the heart of development in the Global South. This intention, however, fell short of having a meaningful impact in the lives of adolescents with disability, evidenced by the invisibility of disability issues in research and public health policies and strategies (Dutta & Chakraborti, 2022; Peta, 2018). Although disappointing, this is not surprising; historically, the sexuality of people with disability has been ignored and suppressed, leading to negative SRH outcomes and contributing to oppression and marginalization (Addlakha et al., 2017; Esmail et al., 2009). The inclusion of people with disability from the Global South in SRH discourse is essential to inform understanding of key issues and needs and to help secure the social positioning of people with disability as equal citizens with rights. The present study begins to address this gap by exploring the SHR of adolescents with cerebral palsy (CP) in rural Bangladesh.

CP is a term referring to a group of disorders that impact a person’s movement and posture and is one of the major causes of childhood disability worldwide (Oskoui et al., 2013; Rosenbaum et al., 2006). Adolescents with CP are reported to experience numerous difficulties with their SRH. For example, adolescents with CP report barriers accessing SRH information and education resulting in adolescents’ being “uninformed” about SRH and having “misconceptions” about how their sexuality may be impacted by impairment, or not (Berman et al., 1999; East & Orchard, 2013). Adolescents with CP also report difficulties in peer and social relationships including delayed dating behaviors and physical and emotional difficulties with sex (Blum et al., 1991; Whitney et al., 2019; Wiegerink et al., 2008). Research with adolescent women with CP has reported gynecologic complaints (Burke et al., 2010) including menstrual issues (Zacharin et al., 2010). Parental anxiety regarding adolescent sexual development is also reported (Zacharin et al., 2010). While there is a growing body of research about the SRH of adolescents with CP in the global north, there is a paucity of research from the Global South (Shah et al., 2022). To date, the literature from the Global South has focused only on topics such as the sexual development of girls with CP (Rao et al., 2019) and the perspectives of caregivers on the pubertal changes of their daughters with CP (Rao et al., 2021).

The dearth of research on the SRH of adolescents with CP from the Global South is striking for two reasons. First, CP is more common and more severe in the Global South (Oskoui et al., 2013; Rosenbaum et al., 2006). In Bangladesh, CP affects 3.4 per 1,000 children, two thirds of whom require (but often lack) wheelchairs for mobility and more than half of whom have cognitive or speech impairments. Moreover, many have epilepsy, communication, visual and/or hearing impairments and 97% live below the poverty line contributing to complex support needs (Khandaker et al., 2019). Second, sociocultural norms impacting SRH differ internationally. In rural Bangladesh, for example, dating among adolescents is uncommon and abstinence prior to marriage is highly valued. Adolescent marriage, however, is a sociocultural norm (Hossain et al., 2016). There is therefore a need for research that accounts for the range of sociocultural contexts in which adolescents with CP live to guide culturally relevant SRH services and support.

The World Health Organization (2002) describes sexuality as a “central aspect of being human” and defines sexual health as “a state of physical, emotional, mental and social well-being in relation to sexuality; it is not merely the absence of disease, dysfunction or infirmity.” However, historically, the sexuality and sexual health of adolescents with disability has been approached from a disease and medical model, focused on areas of risk and dysfunction (Chappell & de Beer, 2019). A sex-positive approach challenges this notion and encourages understanding of SHR as “developmentally normal” (Harden, 2014) and encompassing a broad spectrum of topics pertinent to adolescents’ lives, such as identity and self-esteem, peer and family relationships, and physical, emotional and sexual development, among others (Harden, 2014). Research is needed, from the Global South, that explores the SHR of adolescents with CP, using a sex-positive approach. Framed through the often unheard perspectives of adolescents with CP and their parents, the present study sought to answer the following question: How do adolescents with CP, growing up in rural Bangladesh, experience their SRH?

## Method

This descriptive exploratory study is part of a broader mixed-methods project investigating the well-being of adolescents with CP in rural Bangladesh. Previously, the health-related quality of life (Power et al., 2019) and menstrual experiences of adolescent women with CP (Power et al., 2020) have been reported. The present paper focuses on adolescents’ experiences of their SRH more broadly, and their hopes and desires for the future, including feelings about growing up, psychosocial aspects of sexual development, bodily changes, attitudes to marriage and childbearing, sexual vulnerabilities and access to SRH information and education.

This study has ethical approval from the Bangladesh Medical Research Council (BMRC/NREC/2013–2016/1165) and University of Sydney Human Research Ethics Committee (2016/646). All procedures performed in this study were in accordance with the ethical standards of these institutes and with the 1964 Declaration of Helsinki and its later amendments or comparable ethical standards.

### Participants

A total of 21 focus groups were conducted with 100 participants (24 adolescents with CP and 76 caregivers) over 7 non-consecutive days. Consultation with disability workers and parents of adolescents with CP in the study locale, during the design stage of the present research, identified focus groups as the preferred method of data collection, than surveys or individual interviews, as focus groups would not require literacy and were perceived to be less threatening than individual interviews because participants could better control their level of participation. Focus groups also allowed for discussion between participants which produced a rich depth of understanding, and the facilitators were able to use group dynamics to break the ice when discussing sensitive and taboo topics (Robinson, 1999). Focus groups have been used successfully in previous SRH research with adolescents in Bangladesh (Nahar et al., 2013).

Recommendations on the number of participants in focus groups vary between four and twelve per group (Carlsen & Glenton, 2011). However, groups that include people with cognitive or sensory impairments are recommended to be smaller (Kroll et al., 2007). In the present study, 6 focus groups were conducted with adolescents with CP consisting of 2 to 6 participants each and lasting between 30 to 45 min. For caregivers, 15 focus groups were conducted consisting of 2–7 participants each and lasting between 35 to 60 min. Of the caregivers, 24 were parents or other relatives to the participating adolescent with CP and 52 represented an adolescent with CP who was considered unable to self-report. Social norms dictated that mothers were able to discuss their sons or daughters, whereas fathers only discussed their sons. Caregivers for the female adolescents with CP were mothers (*n* = 30), a grandmother (*n* = 1), an aunt (*n* = 1) and an older sister (*n* = 1), hereafter referred to as mothers. For male adolescents with CP, caregivers were mothers (*n* = 26) and fathers (*n* = 17). Participant demographics are provided in Table 1.

**Table 1:** Characteristics of adolescents with CP

	Adolescents with CP (*n* = 24)	Adolescent with CP represented by parents (*n* = 76) ^a^
	Mean (SD)	Mean (SD)
Age (years)	14.6 (1.7)	14.9 (1.6)
	n (%)	n (%)
Gender		
Female	12 (50.0%)	33 (43.4%)
Male	12 (50.0%)	43 (56.6%)
GMFCS		
Level I	7 (29.2%)	20 (26.3%)
Level II	7 (29.2%)	15 (19.7%)
Level III	7 (29.2%)	8 (10.5%)
Level IV	1 (4.2%)	9 (11.8%)
Level V	2 (8.3%)	24 (31.6%)
Associated impairment		
Speech impairment	8 (33.3%)	52 (68.4%)
Intellectual impairment	4 (16.7%)	43 (56.6%)
Epilepsy	6 (25.0%)	19 (25.0%)
Hearing impairment	2 (8.3%)	8 (10.5%)
Visual impairment	1 (4.2%)	7 (9.2%)
Type of housing		
Kutcha house	17 (70.8%)	55 (72.4%)
Semi-pucca house	6 (25.0%)	12 (15.8%)
Pucca house	4 (16.7%)	9 (11.8%)
Enrolled at school ^b^	17 (70.8%)	21 (27.6%)
Married	2 (2.6%)	2 (2.6%)

*GMFCS*  Gross Motor Function and Classification System; *SD* Standard Deviation. Type of housing: Kutcha = impermanent mud house, semi-pucca = semi-permanent house, pucca = permanent house

^a^n is inclusive of 24 self-participating adolescents with CP; ^b^ has ever been enrolled in education in their lifetime.

Participants were identified using the Bangladesh Cerebral Palsy Register (BCPR). BCPR is the first population-based register of children and adolescents with CP in a low- and middle-income country. The register covers a defined geographical region in the northern part of Bangladesh and holds data on socio-demographic and clinical characteristics of children and adolescents with CP. Children and adolescents with CP are identified for BCPR using Key Informant Methodology described in Khandaker et al. (2015). For the present study, eligible participants were adolescents with CP registered into the BCPR aged 10 to ≤ 18 years (considered a normative classification of adolescence in Bangladesh, (Sigma et al., 2017). Participation was also invited for each adolescent’s primary caregiver classified as a parent, grandparent, aunt, uncle, (older) sibling or other relative who provided majority of care and support.

The parents of eligible adolescents with CP were contacted by phone by a community-worker, and they and their adolescent with CP were invited to participate in focus groups. Due to high rates of illiteracy in the study location information about the focus groups was provided verbally, including the purpose of the focus groups, topics and format. This information was provided again prior to each focus group to ensure understanding and free informed consent. Parents were also given a phone number they could call to ask questions about the interview. Prior to the interview, informed verbal and written consent was obtained for all individual participants. In cases of illiteracy, written consent was obtained by thumbprint. Minors (i.e., 14–18y) provided verbal assent, and their parent or legal guardian provided written consent.

### Procedure

Semi-structured focus group interviews were conducted with adolescents with CP and separately with their primary caregivers between February and March of 2018. Adolescent focus groups were conducted in small groups according to gender, with an interviewer also matched on gender; and similarly, for parents (i.e., matched on caregiver gender rather than child gender). Facilitators were trained in conducting semi-structured focus groups about SRH with people experiencing marginalization.

A semi-structured focus group guide was used. The guide was pilot tested with two groups to confirm the feasibility and was then refined to incorporate local dialect and improve in-depth probing. During each focus group, facilitators built rapport with participants prior to formally commencing the interviews to encourage relaxed interaction and conversation (Nyumba et al., 2018). The facilitators used body language to engage with participants and asked probing questions to facilitate conversation between group members. Observational data were recorded in journal format during the focus groups and reflexive notes made throughout the research process by the first author. This allowed for reflection by the author to understand their subjective positioning in relation to participants experiences and discourse.

Focus groups were conducted in Bengali by researchers for whom Bengali was their day to day language, and digitally recorded. A broad definition of SRH was used drawing on the World Health Organization (2002) working definition of sexuality and Circles of Sexuality model (*see* Dailey (1981)) to explore aspects of SRH pertinent to adolescents lives and that were acceptable to discuss in a group format. Adolescents with CP were asked how they felt about growing up and what they hoped for themselves for the future, knowledge of puberty including physical, emotional and social changes, beliefs, expectations and hopes regarding marriage and having children, and access to sexual and reproductive health information, education and support. Questions were tailored reflective of the gendered makeup of each group. Parents were asked the same questions as well as a question about sexual abuse and disability, and about their own experiences providing care. The facilitator was trained to ensure that personal information that could impact engagement or safety were not shared in the group setting.

### Data Analysis

The interview transcription and translation process have been outlined previously, see Power et al. (2020). Data were analyzed using Braun and Clarke’s (2006, 2019) method for reflexive thematic analysis. The transcripts were initially read and re-read in an ‘active way’ to identify patterns across the data. Coding frames for the adolescent and caregiver data were then developed using a hybrid of deductive and inductive methods. This approach allowed for the research questions to be answered and for the exploration of unexpected themes within the data. Where commonalities in concepts were identified, they were grouped together to create higher-order codes. Adolescent and parent data were then brought together to identify similarities and differences in accounts, and a final coding frame was developed through discussion with team members. Code descriptions were then devised, and data were coded using NVIVO 11 by one member of the research team. Consistency in coding was checked by a senior member of the team. The codes were then re-organized into preliminary themes which were discussed by the team, an iterative process which involved moving back and forth to ensure the themes were embedded in the data. Similarities and differences between adolescent men versus adolescent women with CP were observed in participants’ accounts. All authors read and commented on the preliminary themes, and through a process of discussion the themes were re-organized and refined.

## Results

**Fig. 1:**
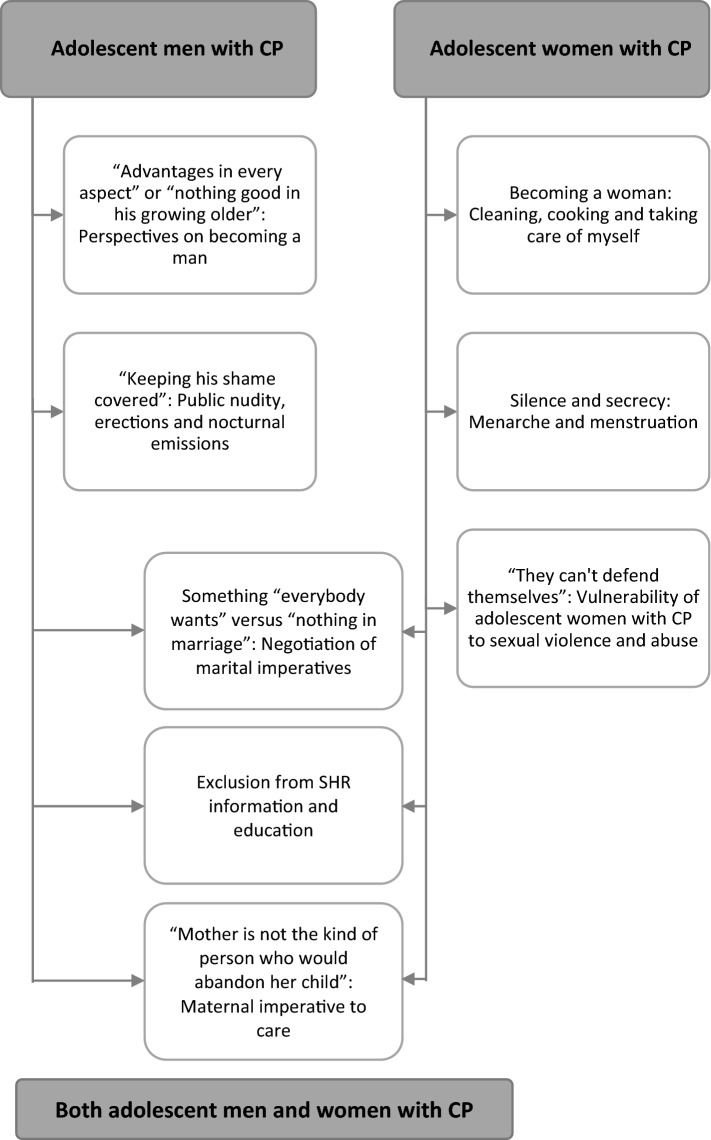
Themes

A total of eight themes were identified, of which two pertained to adolescent men with CP, three pertained to adolescent women with CP and three pertained to both groups, see Fig. 1.

### Adolescent Men with Cerebral Palsy in Rural Bangladesh

#### “Advantages in Every Aspect” or “Nothing Good in his Growing Older”: Perspectives on Becoming a Man

A number of adolescent men with CP described feeling positive about growing up as it brought “advantages in every aspect.” The increased autonomy that came with age allowed these adolescent men to enjoy “hanging out and playing with friends.” Most said that they had at least two or three friends, either from school or neighboring households. Conversely, some adolescent men with CP reported being excluded from social life, “nobody wants to mingle with me”; others said they were bullied and harassed because of their disability “they scold me and say things like ‘leave from here’ and “they call me lengri [handicapped].” A mother told of the impact this had for her son, “if anybody taunts him then he’ll start crying after coming home. He swears and wishes for his death.”

Some men with CP said that now they were adolescents they enjoyed activities such as “studying,” and many had aspirations to “start working and become financially independent.” These adolescent men said they were hopeful about the future, as one commented, “my parents were very worried about me when I was a child. Now I’m thinking of completing my studies and doing something in my life!” More often, however, adolescent men with CP lacked opportunities. Many parents expressed concerns about their son’s future and said they felt “worry,” “fear,” “unhappiness” and “exhaustion.” The mother of an adolescent man with CP said, “we find nothing good in his growing older. We always say that it would be good if Allah takes him away sooner than later then he would relieve us from pain and suffering.” These accounts provided insight to the opportunities and restrictions experienced by adolescent men with CP growing up in rural Bangladesh.

#### “Keeping his Shame Covered”: Public Nudity, Erections, and Nocturnal Emissions

As adolescent men with CP went through puberty and their bodies matured, new expectations for modesty ensued including “keeping their shame covered.” A number of parents described difficulties teaching their sons bodily privacy, as the mother of an adolescent man with CP commented, “my son does not understand anything… he touches his genitals and shows it to our neighbours.” Another mother commented, “we keep his shame covered as much as possible but at times he goes naked. When I try to cover him, he screams and says he does not want it.” Parents said that caring for their son while also considering other women in the household could be very difficult, as a mother explained, “my son is 15 and my daughter is 10, I keep my daughter separate from my son. This is because my son is physically incapable to cover himself and hide his shame.” Adolescent men’s nudity also caused tension within the local community, as a father told us,

My son would wear good clothes and trousers but at times he gets bothered with long pubic hair, so he takes off his clothes and becomes nude - by himself. The neighbours tell us off, that it is a sin to keep him nude like this, we ought to cover him. But we always try to keep him covered!

Some adolescent men with CP were also reported to lack discretion managing bodily experiences such as erections and nocturnal emissions. Ejaculation from nocturnal emissions was discussed by adolescent men with CP and fathers as harmful to health and was said to cause issues such as “headaches,” “eyes losing sight,” “becoming weak” and “turning ugly.” A father expressed concerns that, “if this happens too often [ejaculation], they will stay in bed and will not be able to walk.” Numerous fathers said they sought to “cure” their sons from having nocturnal emissions by visiting the Kabiraj (traditional healer). A father explained, “my son used to have wet dreams. I’ve taken him to a doctor and put an end to this. I fed him herbal medicine.” An alternative “fix” was for adolescent men with CP to become married; however, marriage was not an option available for all adolescent men with CP, as discussed later in this paper. The father of an adolescent man with CP said, “this illness gets cured when you get your son is married but we can’t do that. We neither ask him about nor look for girls to get him married. Now his hair has turned grey.” Difficulties adhering to the social norms and requirements for their age and developing sexual bodies contributed to the exclusion of adolescent men with CP and created additional layers of stress and concern for parents.

### Adolescent Women with Cerebral Palsy in Rural Bangladesh

#### Becoming a Woman: Cleaning, Cooking, and Taking Care of Myself

Unlike some of their male peers who were developing independence and forging life outside of their home, adolescent women with CP described this life stage as involving increased restrictions. Adolescent women discussed taking on new domestic tasks as they grew up such as “dusting,” “tidying up the table,” “cleaning up the house” and “cooking.” Due to their physical impairments, many participants relied on support from other women in their household to complete these tasks, however, taking part in these activities made many participants feel useful as members of their households. Others did not like these responsibilities and the increased regulation that was imposed on them as they grew older. An adolescent woman with CP complained, “people play in the field in their childhood, but I’m not allowed to anymore. My friends play, I watch them” and another said, “I used to play and roam around before but now I’m not allowed to do neither of them.”

Taking pride in appearance emerged in the narratives of adolescent women with CP and their parents as an important way of taking responsibility for oneself. An adolescent woman with CP said, “I’ve grown up so I always take care of myself. I keep my hair tied. I go to school and take a shower after returning home.” Parents discussed appearance as being an important part of how their daughter with CP presented to the world, a mother explained, “we keep them well dressed. We do not want others to look down at them. If they look good, they will feel good at heart.” These accounts provided insight to the experiences and expectations placed on adolescent women with CP during this life stage regarding physical appearance and domestic roles.

#### Silence and Secrecy: Menarche and Menstruation

A number of adolescent women with CP described menstruation as uncomfortable and distressing as it caused “cramps,” “pain in my lower abdomen” and a “severe stomach-ache.” Some adolescent women used medicine to help manage these discomforts, as a mother said, “my daughter cannot speak. She screams. I calm her down saying different things. I keep chanting Allah’s name. Then, I give her medicines when she gets acidity.” However, pain relief and contraception to reduce or regulate menstrual symptoms were often frowned upon, as another mother said, “The doctor forbade me to give her any medicine.” A number of adolescent women reported signs of vaginal infections, such as colored discharge, however, said they didn’t have anyone they could speak to for support or advice, “my mom doesn’t know.” Adolescent women with CP lacked access to formal information and education about their menstruation and were often reliant on peers for information, “I only share those things with someone next door, someone of my age.” Most said that they would not speak to their mother about menstruation, although one adolescent woman said, “I talk to my mother, but I cannot say everything.” Adolescent women with CP and their mothers discussed menstruation as a sign that girls were “mature.” However, this was also perceived to be associated with increased vulnerability to sexual abuse, causing some mothers to pursue contraception for their daughters: “they give her vaccines for 3 months as they are women now and can be attacked by men.”

#### “They Can’t Defend Themselves”: Vulnerability of Adolescent Women with CP to Sexual Violence and Abuse

[Due to the sensitivity of the topic, the following information was discussed by parents only]. Although a handful of parents stated that they “haven’t heard of anything like that [sexual assault] happening to disabled children,” the majority of parents said that “disabled people are always subjected to some form of neglect” and that “disabled children are vulnerable [to sexual violence] as they can’t defend themselves.” Parents were particularly concerned for the safety of their daughters who had limited physical mobility and/or were unable to communicate verbally. A mother of an adolescent woman with CP explained:

What scares me is that my daughter won’t be able to tell me if anything happens to her. She won’t be able to call anybody for help. I have to keep her alone at home but anyone could come and take advantage of her.

These concerns resulted in restrictions being imposed on adolescent women with CP, such as not being allowed out unaccompanied, as a mother commented, “my daughter is very beautiful. She always stays inside the house. This kind of incident happens a lot, this is why I don’t let her out.” However, leaving daughters home unaccompanied was also a source of anxiety, a mother explained, “in my opinion, anything can happen to those who have daughters at home. I feel anxious thinking about my daughter when I’m outside.” Fear of sexual violence was reinforced by experiences of physical violence. The mother of an adolescent woman with CP told us “there were times when my daughter stood before someone outside the house and that person pushed her to the ground. Sometimes she got beaten by others.” These experiences created a cycle of fear that contributed to the isolation and vulnerability of young adolescent women with CP.

### Both Adolescent Men and Women with Cerebral Palsy in Rural Bangladesh

#### Something “Everybody wants” versus “Nothing in Marriage”: Negotiation of Marital Imperatives

The majority of adolescents with CP said they desired to be married and have children because “my friends have gotten married,” “it is obligatory, it should be done,” and “because we can’t stay like we are, we’ve grown up.” Parents often reinforced marital imperatives, as a mother of an adolescent woman with CP commented, “a girl’s life is dedicated to that thing [marriage]. If she doesn’t have that then her life is worthless. Her life will be ruined.” Although most participants discussed marriage as something that “everybody wants,” a small number of adolescent women with CP resisted marital imperatives for reasons such as wanting to “complete education” and that “there is nothing in marriage.” An adolescent woman with CP who wanted to study to become a Doctor explained that getting married would be a barrier to her future aspirations, as “it’s troublesome to get married being a doctor, I’ll have to listen to my husband.”

Other adolescents with CP were unsure if they could get married as “some people with disability can, others can’t.” For example, men needed to be able to earn money to “take responsibility for their wives” and women needed to be able to manage their households. Adolescent women CP said they felt assured that they could get married and have children when they knew of other women with similar impairments who had achieved this. However, many participants were likely to be precluded from marriage due to their impairments. The mother of an adolescent man with CP questioned, “if my son gets married who will provide food for his wife?” She explained that “only one person earns a living in my home and he feeds seven other persons. If my son gets married who will provide for him and the adolescent bride?” For some families, financial resources were adequate to negotiate these barriers. For example, a father said that he might be able to find a wife for his son with CP, “if I offer them property and don’t take dowry” and another said, “if my son wants to get married then I will get him married but I’ll have to take care of their expenses as my son can’t.” Other parents said that marriage would be “impossible” for their child, as “no one will get married to someone who can’t move.” The mother of an adolescent man with CP explained:

If his pillow falls on the floor, from under his head, he is incapable of picking up the pillow and putting it back under his head. At night he wakes up three or four times, he may throw away his cover and I have to get up and put the blanket back on him. He cannot even do this, so how can he get married? I am always worried at heart.

Numerous mothers said they did not want their daughters with CP to become married as they were afraid their daughter would be beaten or abandoned in marriage due to their disability. The mother of an adolescent woman with CP said, “I won’t get her married. If she gets married, everyone will beat her. She cannot speak or hear, so everyone will beat her.” Parents who did not think their child could get married were concerned about what would happen later in life, “there will be a lot of problems when we won’t be around. There isn’t any end to these problems. I worry about his future after I’ll be gone. How will he survive?” These accounts demonstrate the complexities for adolescent women and men with CP as they negotiate cultural requirements for marriage alongside personal desires and ableist exclusions.

#### Exclusion from Sexual and Reproductive Health Information and Education

Adolescents with CP said they found out SHR information from a range of sources. For example, when asked how he knew about puberty an adolescent man with CP said, “I hear my friends talk when I hang out with them” and another said, “I learnt about the physical changes from the physical education classes we used to have in the 7th grade.” Adolescent women with CP said they might speak about it with their “sister-in-law” or “grandma.” However, many adolescents lacked a peer network and the majority of adolescents with CP were not enrolled at school resulting in their exclusion from these information sources. Many parents dismissed their adolescent’s capacity to understand or learn about SRH matters, “she cannot tell. Allah hasn’t given her the strength’ and ‘he is disabled. He won’t understand.’ A number of mothers said that although their daughter had “grown older in age,” it was “no good to think of them as grown up. They are small children as they cannot do anything.” Furthermore, some parents believed their adolescent was diminished in capacity to experience a full range of human emotion. The father of an adolescent man with CP said, “if he was a normal child, then he would have understood everything by now. He is impaired and cannot feel anything that deeply.” These attitudes and beliefs infantilized adolescents with CP and contributed exclusion from SHR information and education.

#### “Mother Is not the Kind of Person Who Would Abandon her Child”: Maternal Imperative to Care

Parents described difficulties as their sons and daughters with CP matured physically but failed to adhere to sociocultural norms and expectations for their age group. Mothers provided the majority of caregiving to their adolescent children with CP, however, said that as their children had gotten older, involvement in intimate aspects of care, such as toileting, bathing and dressing, had become “painful” and “shameful.” This was particularly the case for mothers of adolescent men with CP as a mother explained, “I have shame of my own. You know I feel ashamed. It can be done [caretaking] when it is a girl, but I have a boy. I feel ashamed.” Mothers explained that the responsibilities of caregiving could be too much and that they “wanted to run off.” However, they resisted this due to a maternal imperative to care, as a mother said: “Mother is not the kind of person who would abandon her child. He was in my womb and a mother cannot abandon her child. Others can ignore him, but I cannot do this.”

## Discussion

This study provided insight to the SRH experiences of adolescents with CP in rural Bangladesh, and their parents providing care. For a small number of adolescents with CP, this life stage was positioned positively; adolescents described feeling good about growing up and new opportunities prevailed. However, for most, growing up with CP was framed negatively, marred by increased restrictions, exclusions and vulnerabilities due to ableist assumptions about their impairments and capacities. Both adolescent men and women with CP reported tensions navigating their impairments alongside gendered sociocultural norms that concorded with their sexual development. Mothers often assumed responsibility for the care needs of their adolescent children with CP. However, maternal involvement in their child’s care after puberty disrupted gender and sexuality norms and was a source of distress for many mothers, confirming previous research (Rao et al., 2021).

Adolescent sexuality is governed by complex, often unspoken, social and gender norms (Pulerwitz et al., 2019). In the present study, some adolescent men with CP did not adhere to social norms to keep their pubertal bodies and bodily functions concealed. While many people learn these rules and how to navigate them through observation and interaction with others, some people require direct support and education (Tarnai, 2006). However, the adolescents with CP in our study did not have access to any formal SRH education, were unable to speak with the adults in their lives about these experiences and lacked informal learning from peer networks. These exclusions combined with a lack of private spaces within their homes and the involvement of others in their personal care meant that adolescents with CP were unable to discretely navigate their sexual bodies, contributing to their “sexual marginalization” (Addlakha et al., 2017, p. 5) and perpetuating damaging and incorrect myths such as that people with disability have ‘deviant’ sexualities that need to be controlled and suppressed (Alexander & Taylor Gomez, 2017).

In the present study, both adolescent men and women with CP experienced regulation of their sexual bodies in ways that differentiated them from their peers without disability. In the study location, erections and ejaculation were considered bad for adolescents’ health. However, some adolescent men with CP were unable to discretely manage these bodily experiences resulting in them being given herbal medicine to prevent occurrence. For adolescent women, menstruation was considered good for health and use of contraceptive methods to regulate menstrual cycles was discouraged. However, adolescent women with CP who had high support needs were given contraception due to fears they may become pregnant if sexually assaulted. These responses to managing adolescents’ bodily experiences and risks of sexual violence fail to address underlying concerns and risks and threatens adolescents’ bodily autonomy and SRH rights (Addlakha et al., 2017).

Our findings extended previous research exploring attitudes and beliefs about marriage among adolescents in Bangladesh (Hossain et al., 2017). Similar to adolescents without disability, adolescents with CP discussed marriage as an important life event. Marriage was considered central to well-being, and many people believe that women’s social status is earned through marriage (Hossain et al., 2017). Although decreasing, adolescent marriage in rural Bangladesh is still relatively common; just over three quarters (78.2%) of adolescent girls will be married before 18 years old (Hossain et al., 2016). Adolescents with CP in our study appeared to be protected from traditional marital practices. Only two adolescents, both males, were married. However, this is a complex issue; adolescents with CP were infantilized and positioned as undesirable, unfit and incapable of being husbands and wives and may be excluded from marriage entirely. Marriage offers important social and financial protection, particularly for women, who may face severe social marginalization and financial vulnerability if unmarried as older women (Quinn et al., 2016). However, women with disability who are married report high risks of physical and sexual violence including family beatings and are vulnerable to ‘abandonment’ which brings shame to their family further compromising their safety (Hasan et al., 2014).

### Practice and Research Implications

This research has provided new insight about the SRH of adolescents with CP in rural Bangladesh. There are several practice and research implications of our findings: First, SHR education needs to be sensitively incorporated as part of a life-course approach within services for adolescents with CP in rural Bangladesh. Adolescents with CP in rural Bangladesh are often not at school and are consequently excluded from school-based SRH education programs (Khandaker et al., 2019). Adolescents with CP may also have unique SHR education needs; many adolescents struggled to understand their bodily changes and changed social expectations resulting in socially unaccepted behavior. Explicit learning of social rules about private body parts and behaviors in adolescence is required (Tarnai, 2006). Some adolescents with CP may be sexually active; it is important for education to address these experiences (Sharma & Sivakami, 2021).

Second, parent interventions are required to improve parent–child communication. Adolescents with CP often lacked understanding of their SRH and were unable to discuss their experiences with the adults in their lives. It is important that adolescents can discuss bodily changes, menstruation, relationships, marriage, safe and unsafe touch and experiences of violence (Sharma & Sivakami, 2021). Parent focused interventions to address shame caused by involvement in adolescents intimate care after puberty are also required. Parent-to-parent counselling facilitated within support groups for parents of children with CP (Palit & Chatterjee, 2006) has demonstrated positive outcomes in India helping parents better understand the needs of their child. This low-cost strategy may have utility for rural Bangladesh.

Third, parents, teachers, disability and health professionals, NGO and social workers need to be aware of the potential vulnerability of adolescents with CP to sexual violence and abuse. Mechanisms for responding to abuse that prevent further trauma for adolescents with CP are required. Fourth, adolescents with disability are often omitted from SRH research and public health policies, strategies and budgets (Dutta & Chakraborti, 2022; Peta, 2018). There is a need for adolescents with disability to be included to facilitate strategic and effective responses that improve SRH.

### Strengths and Limitations

There are strengths and limitations to this study. This study is the first to explore the SRH of adolescents with CP in Bangladesh, and among only a small number in the Global South more broadly. While discussion of adolescent SRH in a rural locale of a Muslim country could be presumed to be met with resistance, this was not our experience. Willingness to participate in the present research highlighted the relevance of investigations and the absence of other opportunities for adolescents with CP and their parents to discuss SRH matters. Parents, in particular, used the focus group as an opportunity to seek and share advice with each other.

A central tenet of the present research was to center the experiences of adolescents with CP as people with disability are rarely asked about their SRH experiences, hopes and desires, as it is assumed they have none (Alexander & Taylor Gomez, 2017). By placing adolescents with CP at the center of analysis, we aimed to hear these perspectives and avoid over simplified narratives that problematized adolescent SRH. However, we were limited with this approach as our research methods were inaccessible for adolescents with CP and higher support needs such as those labelled with intellectual and/or communication impairments; instead, parents reported on behalf of these participants. Parents often focused on SRH-related issues and problems. While these were important perspectives, there is also a need for research that enables direct engagement with adolescents with CP and higher support needs. Research using participatory methods that are inclusive for people with higher support needs is required so that these perspectives are adequately represented in research.

We utilized group rather than individual interviews or surveys as focus groups are reported to be a valuable method for the conduct of research about SRH topics with adolescents (Hyde et al., 2005) and pre-study consultation identified this to be a preferred method for the research location. However, we experienced challenges with this approach. The logistical coordination was difficult; participants were invited to arrive for defined times; however, the research coincided with harvest season resulting in some arriving early and others late or not at all. Quick reorganizations of focus groups were required resulting in uneven and at times small group sizes. In addition, although focus groups can enable rich and thick discussions produced by group dynamics, this can also limit in-depth exploration of topics and the telling of personal accounts due to considerations such as lack of anonymity or shyness (Nyumba et al., 2018). To manage these considerations, the facilitators adopted specific strategies to create safe and comfortable environments and build rapport. Notably, the focus groups were conducted in small groups (in a circle format) in private rooms and the facilitators were educated on what topics were acceptable to discuss with each of the groups. With adolescent participants, repeated permission was offered to speak openly, acknowledging a break from traditional adult and adolescent dynamics. Future research embedded in participatory methods may help to further build rapport and enable deeper understanding of adolescents’ experiences.

### Conclusion

In conclusion, this study found that adolescents with CP in rural Bangladesh experience numerous SRH tensions as they transition into their adult lives. Gendered sociocultural norms and ableist assumptions shaped adolescents’ experiences of their SRH. SRH is a central component of adolescent wellbeing, including for adolescents with CP; address of the specific SRH needs of adolescents with CP, and their parents providing care, is crucial to improve outcomes for this population. These findings emphasize the need for disability, health and education services in rural Bangladesh to adopt a life-course approach that incorporates the SRH of adolescents with CP and for the provision of SRH education and support that addresses the physical, cognitive and social needs of adolescents with CP and their parents providing care.

## Data Availability

These data are not available on request as the data contain potentially sensitive and identifying information.
